# CT imaging features of atrioventricular shunts: what the radiologist must know

**DOI:** 10.1007/s13244-015-0452-7

**Published:** 2015-12-05

**Authors:** Simon Nicolay, Rodrigo A. Salgado, Bharati Shivalkar, Paul L. Van Herck, Christiaan Vrints, Paul M. Parizel

**Affiliations:** Department of Radiology, Antwerp University Hospital, Wilrijkstraat 10, 2650 Edegem, Antwerp Belgium; Department of Cardiology, Antwerp University Hospital, Edegem, Belgium

**Keywords:** Congenital heart disease, Atrial septal defects, Ventricular septal defects, Cardiac CT, CT angiography

## Abstract

**Abstract:**

In the last decade, cardiac computed tomography (CT) has gained mainstream acceptance for the noninvasive exclusion of significant coronary disease in a selected population. Improvements in electrocardiogram (ECG)-triggered imaging techniques also allow, by extension, a proper evaluation of the complete heart anatomy. Given the increasing worldwide clinical implementation of cardiac CT for coronary artery evaluation, radiologists can, incidentally, be confronted with unfamiliar and previously unsuspected non-coronary cardiac pathologies, including congenital morphological defects. This presence of congenital heart disease (CHD) should not be overlooked, being the most common form of birth defect, with a total birth prevalence of 9.1 per 1000 live births worldwide [1]. The prevalence of adult patients with CHD is estimated to be 3000 per million adults [2]. Ventricular septal defects (VSDs) are the most frequent subtypes of CHD, accounting together with atrial septal defects (ASDs) for nearly half of all CHD cases [1]. While some small defects are rarely symptomatic and can go undetected for life, others are clinically significant and require adequate and timely medical intervention. In this article, we present the CT imaging features of atrioventricular (AV) shunts, highlighting both their embryological origins and associated relevant clinical features.

**Teaching points:**

• *Congenital heart disease (CHD) is the most common birth defect.*

• *Ventricular and atrial septal defects account for nearly half of CHD cases.*

• *Atrioventricular defects can frequently be detected on a cardiac CT.*

• *Radiologists must be able to identify clinically significant atrioventricular defects.*

## Introduction

In the last decade, cardiac computed tomography (CT) has gained mainstream acceptance for the noninvasive exclusion of significant coronary disease in a selected population. Improvements in electrocardiogram (ECG)-triggered imaging techniques also allow, by extension, a proper evaluation of the complete heart anatomy. Given the increasing worldwide clinical implementation of cardiac CT for coronary artery evaluation, radiologists can, incidentally, be confronted with unfamiliar and previously unsuspected non-coronary cardiac pathologies, including congenital morphological defects. This presence of congenital heart disease (CHD) should not be overlooked, being the most common form of birth defect, with a total birth prevalence of 9.1 per 1000 live births worldwide [1]. The prevalence of adult patients with CHD is estimated to be 3000 per million adults [2]. Ventricular septal defects (VSDs) are the most frequent subtypes of CHD, accounting together with atrial septal defects (ASDs) for nearly half of all CHD cases [1]. While some small defects are rarely symptomatic and can go undetected for life, others are clinically significant and require adequate and timely medical intervention. In this article, we present the CT imaging features of atrioventricular (AV) shunts, highlighting both their embryological origins and associated relevant clinical features.

## Concise overview of relevant embryology and anatomy

**Fig. 1:**
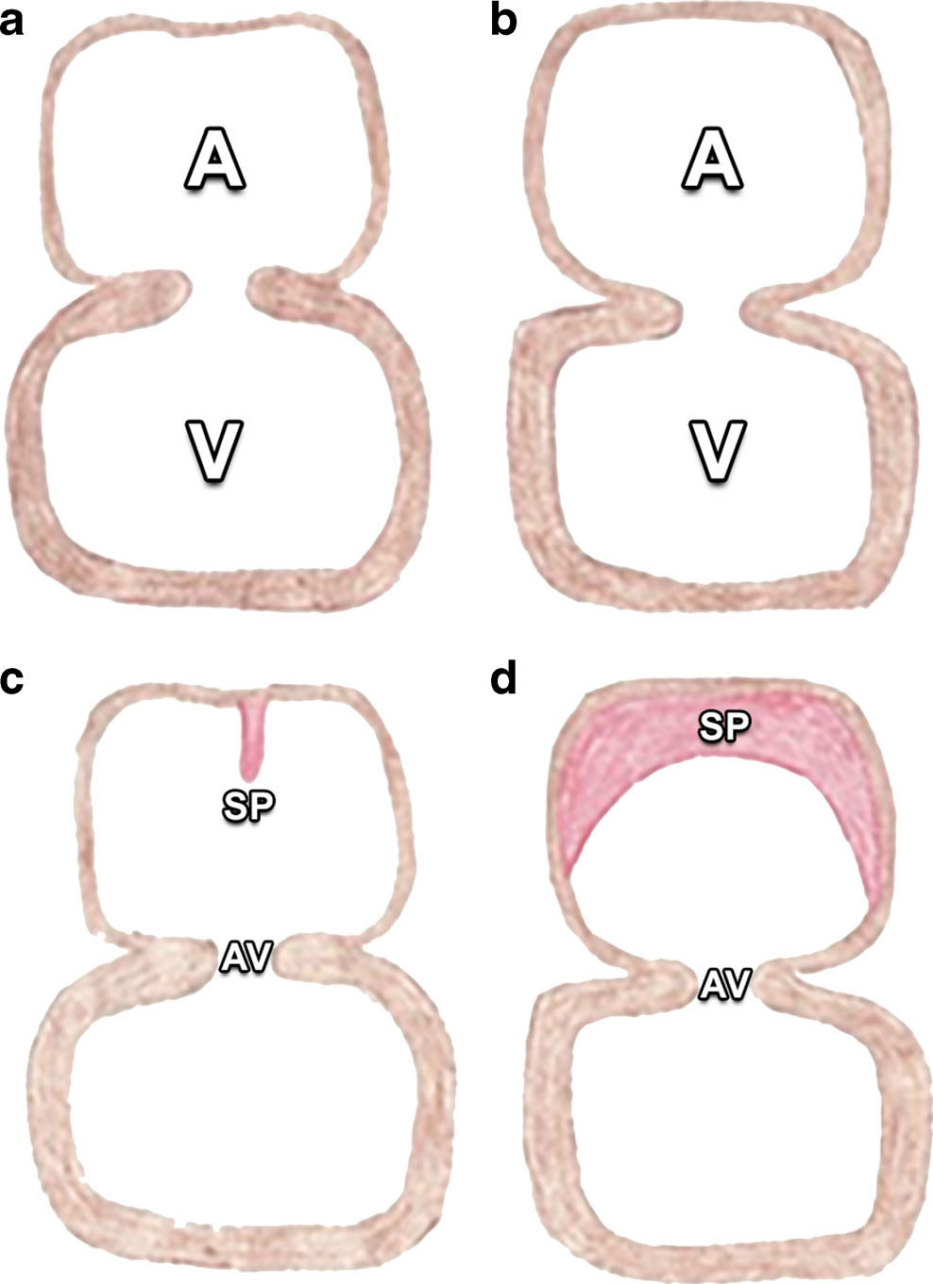
Primitive atrium and ventricle before the age of 30 days in a frontal view (**a**) and lateral view (**b**) and around 30 days in a frontal view (**c**) and lateral view (**d**). Before the age of 30 days, the embryonic heart is composed of a primitive atrium (A) and ventricle (V) without a dividing septum. Around 30 days of gestation the primary atrial septum or septum primum (SP, drawn in *red*), begins to grow as a crescent-shaped structure downwards from the roof of the primitive atrium toward the atrioventricular (AV) canal

**Fig. 2:**
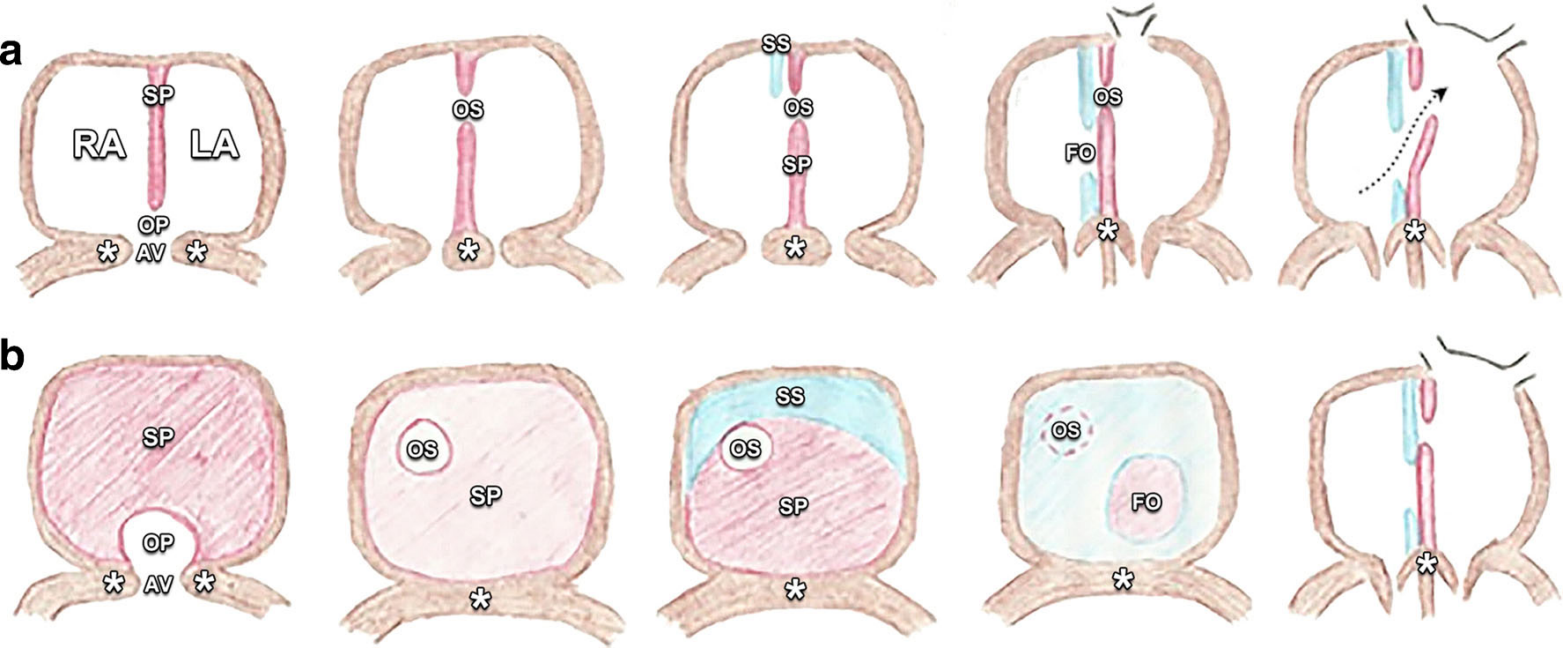
Evolution of atrial septation in afrontal view (**a**) and lateral view (**b**). The fifth drawing of panel **b** is also a frontal view. The septum primum (SP, drawn in *red*) grows towards the developing endocardial cushions (*asterisk*) at the level of the atrioventricular (AV) canal, dividing the primitive atrium into what will become the left atrium (LA) and right atrium (RA). An opening, called the ostium primum (OP), is left between the edge of the SP and the endocardial cushions at the AV canal. The OP allows oxygenated blood from the placenta to bypass the lungs and flow between the developing atria. During further development, the SP will gradually fuse with the endocardial cushions. However, before closure, a second opening in the SP will appear. This opening, known as the ostium secundum (OS) allows continuing interatrial communication. At the age of 36 days, a second septum, the septum secundum (SS, drawn in *blue*) appears from the atrial roof immediately to the right of the SP. In its course, it overlaps the OS and eventually fuses with the endocardial cushions. This septum, too, leaves an opening called the foramen ovale (FO). The superior edge of the SP partially regresses. The inferior part acts as a valve, allowing only right-to-left flow through the FO, as prenatal blood passes from the right atrium to the left atrium (*dotted arrow*) since right atrial pressure exceeds left atrial pressure. After birth, increased pulmonary return will raise the left atrial pressure, which will cause the valve to functionally close, as shown in the fifth drawing of Figure **b**, and eventually fuse with the septum secundum at the age of 6 months

**Fig. 3:**
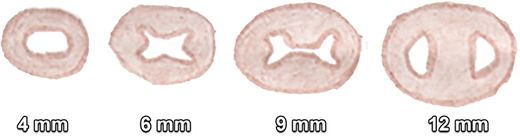
Development of the AV canal at the embryological stages of 4, 6, 9 and 12 mm. The endocardial cushions proliferate in different planes, not only dividing the AV canal in a left mitral and right tricuspid canal but also forming part of the membranous ventricular septum and closing the atrial OP

**Fig. 4:**
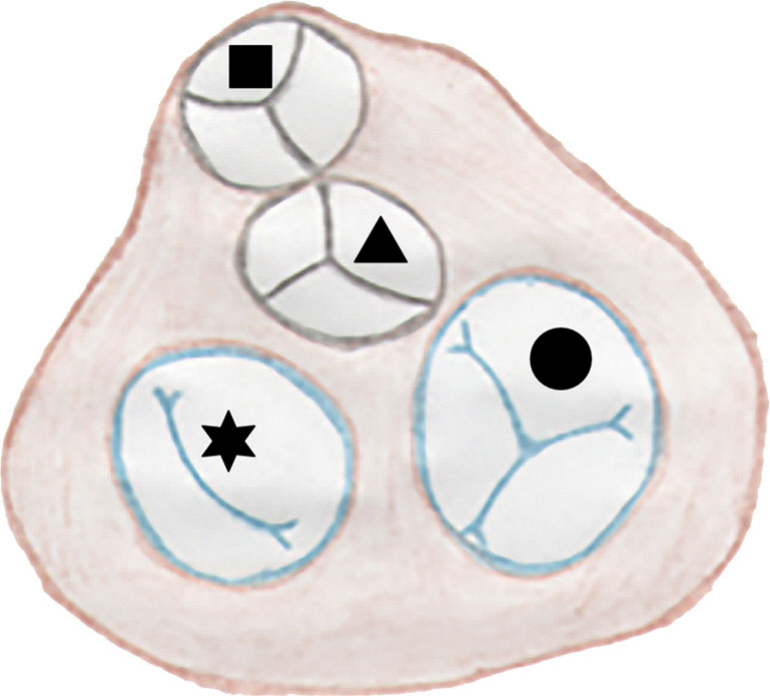
Normal anatomy of the AV canal. It is divided into a right canal with a tricuspid AV valve (*circle*) separating the right atrium and the right ventricle and a left canal with a bicuspid (*mitral*) AV valve (*star*) separating the left atrium and the left ventricle. The aortic valve (*triangle*) and the pulmonary valve (*square*) are located anterior of the AV valves

**Fig. 5:**
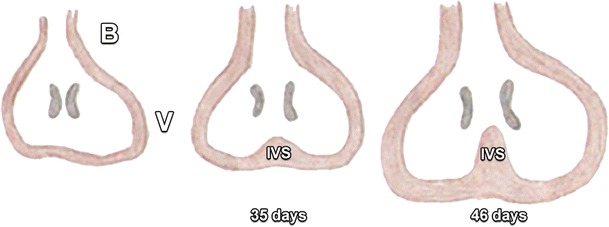
At the end of the fourth week the unseptated primitive ventricle (V) is connected on one side with the primitive atrium (which is not shown and lying directly behind of the primitive ventricle on this drawing), through the atrioventricular (AV) canals. On the other side it is connected with the bulbus cordis (B). The bulbus cordis consists of the conus cordis and the truncus cordis and will give rise to the future aorta and truncus pulmonalis. In the fifth week the left and right part of the primitive ventricle start to grow, creating a median muscular ridge (interventricular septum, IVS). Initially, this ridge mainly results from the joining of the growing ventricles on each side. In a second stage, cell growth from the ventricular septum itself contributes to the size. The primitive interventricular septum grows up to the endocardial cushions, creating two chambers. However, it will stop growing before it reaches the endocardial cushions, leaving an opening that allows interventricular communication, called the interventricular foramen

**Fig. 6:**
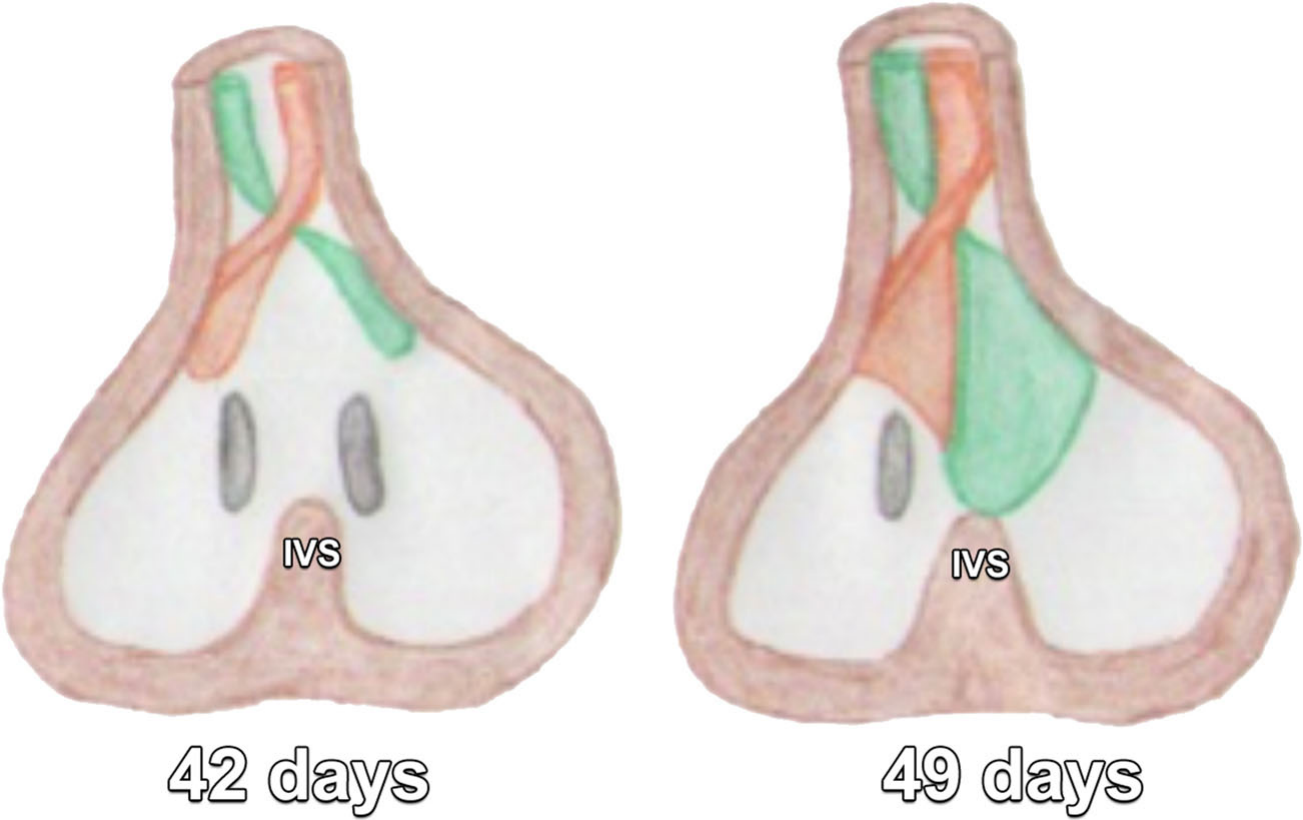
In the fifth embryological week, two ridges (drawn in *orange* and in *green*) develop in the wall of bulbus cordis. These ridges will converge in the midline and grow and extend toward the muscular interventricular septum (IVS), in a helical way. This leads to the formation of the aortopulmonary septum, which divides the bulbus cordis into two arterial channels, the aorta and the truncus pulmonalis, the former continuous with the left ventricle and the latter with the right ventricle. At the end of the seventh week, the ridges fuse with the atrioventricular (AV) endocardial cushions and with the muscular septum, forming the membranous septum and closing the interventricular foramen

**Fig. 7:**
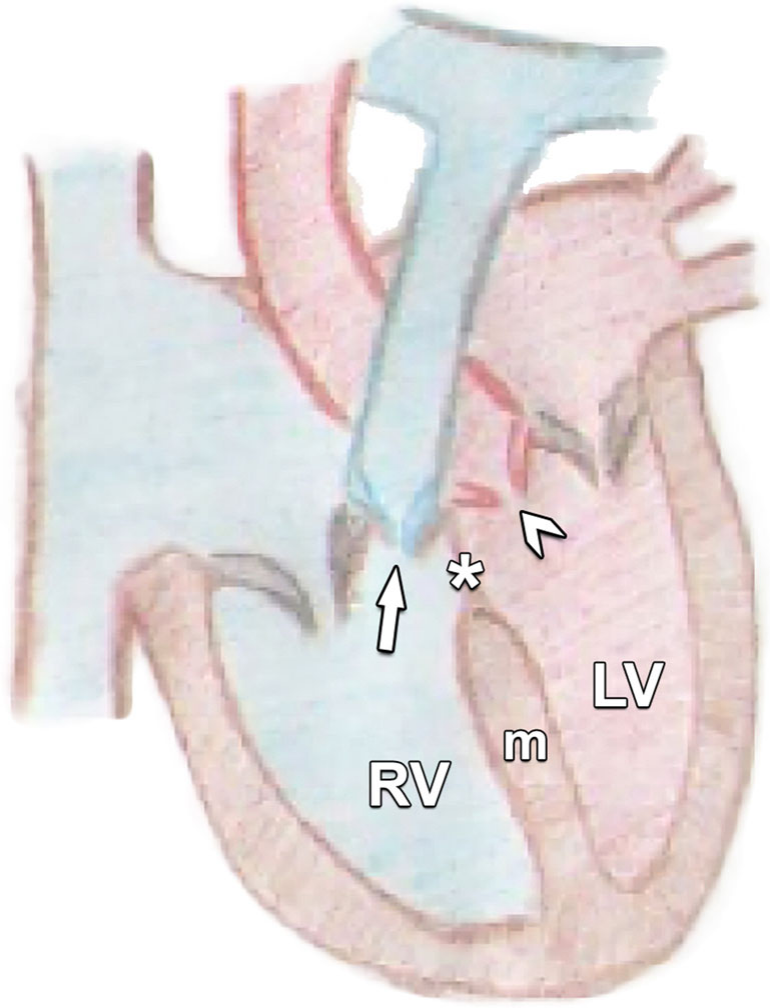
Normal anatomy of the interventricular septum. It consists of a thick muscular portion (m) and a thin oval-shaped membranous portion (*asterisk*). The latter lies immediately inferior to the aortic valve cusps (*arrowhead*) and the pulmonary valve cusps (*arrow*). *RV* right ventricle, *LV* left ventricle

The development of atrial septation initiates 28–30 days after gestation, with key elements of this development shown in Figs. 1 and 2 [3–5]. In short, the AV canal develops together with atrial septation. Cells from the primitive endocardium migrate and form the endocardial cushions, which are swellings of connective tissue surrounding the AV canal (Figs. 3 and 4) [4, 5]. Septation of the primitive ventricle occurs simultaneously with atrial septation in the fifth to seventh week. Furthermore, it is closely related to the septation of the truncus and conus cordis, which leads to the formation of the future ventricular outflow tracts. The complete separation of both ventricles is the final result of these two processes (Figs. 5, 6, and 7) [4, 5].

## Multimodality imaging of intracardiac shunts

The most used technique for intracardiac shunt evaluation is transthoracic and especially transoesophageal echocardiography, which is by many still considered the reference standard [6]. However, both CT and magnetic resonance imaging (MRI) can also be used to visualize intracardiac shunts [7, 8]. The choice between these imaging modalities often depends on the local expertise and available equipment. When further comparing CT versus MRI, MRI is a more complex technique but may provide functional cardiac information and may be able to quantify the degree of an AV shunt by volumetric measurements of the ventricles and flow quantification over the cardiac valves.

Furthermore, MRI has the advantage of not using radiation and can also be performed without the use of intravenous contrast administration. Conversely, CT has been proven to deliver exquisite anatomic visualization of cardiac anatomy in a short examination time with little physician input. While it is not customary to use CT for the visualization of intracardiac shunts, the use of ECG gating techniques during routine examinations targeted at the coronary arteries allows excellent simultaneous visualization of these congenital defects, as such, enhancing the value of the radiology report [8, 9]. When large, they can also be detected on non-ECG-gated CT examinations.

## Atrial shunts

An ASD is defined as a persistent opening in the interatrial septum after birth, allowing communication between the left and the right atrium [5, 6]. When present, oxygenated blood shunts from the left atrium to the right atrium, the shunted volume depending on the size of the defect.

The reported birth prevalence of ASDs is 1.64 per 1000 live births, accounting for 13 % of all CHDs [1]. They account for 25–30 % of CHD cases diagnosed in adulthood [10]. Remarkably, most ASDs, even those with a large left-to-right shunt, go undiagnosed through most of one’s childhood and may only become symptomatic in the fifth decade or later. The exact age at which symptoms begin is highly variable and not exclusively related to the size of the shunt. With advancing age, the risk of developing exercise intolerance, atrial fibrillation or even decompensated right heart failure and pulmonary hypertension increases [11].

ASDs are detectable on both contrast-enhanced CT and MR examinations. While they can be appreciated on standard axial images, their presence and further characterization is often better appreciated on oblique, reformatted images perpendicular to the atrial septal plane.

### Patent foramen ovale

During fetal life, a normal interatrial communication through the oval foramen exists, providing oxygenated blood from the inferior vena cava into the left atrium. As such, this communication is not a true ASD but a necessary passage tunnelled during embryologic development. It is commonly encountered in newborn babies. With advancing age, left atrial pressure increases, functionally closing the foramen ovale by pushing the septum primum flap against the septum secundum. In a vast majority of cases, normal development evolves to complete septal fusion. However, a failed fusion with persistent interatrial communication is called a patent foramen ovale (PFO), a common finding found in up to 25 % of adults [12].

**Fig. 8:**
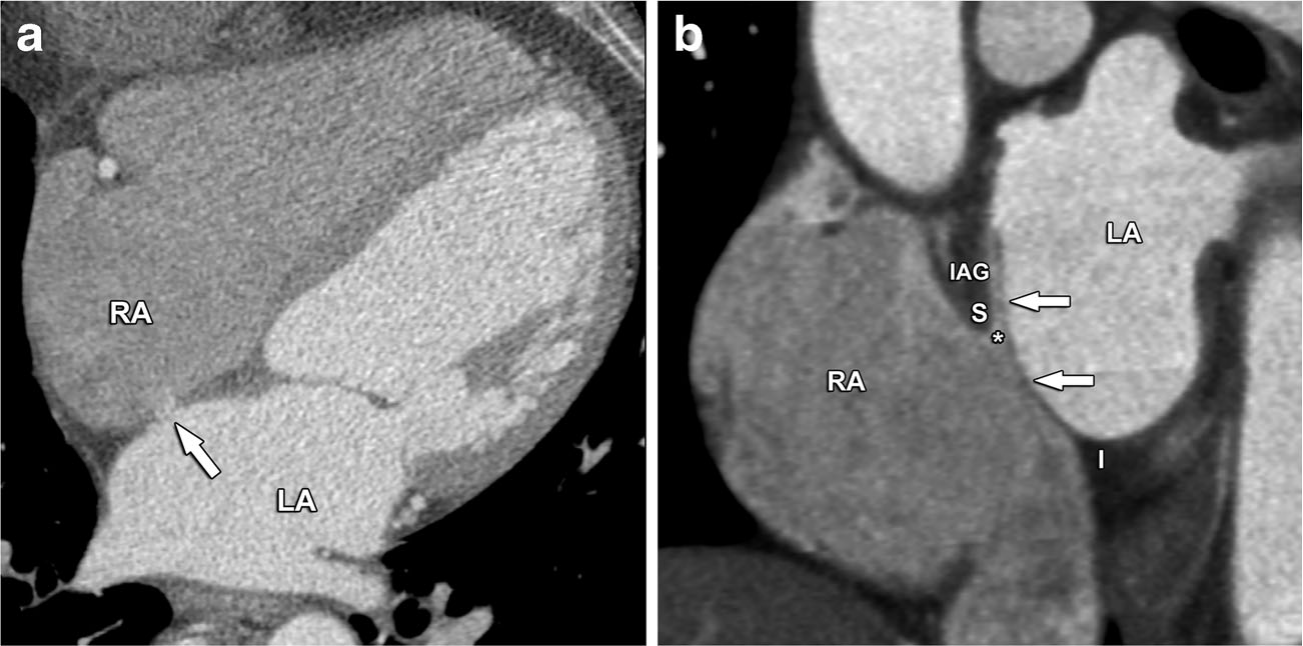
Four-chamber contrast-enhanced CT image **a** illustrating a small patent foramen ovale (*arrow*). Oblique coronal contrast-enhanced CT images **b** show the interatrial septum at the level of the fossa ovalis. A patent foramen ovale (*asterisk*) is the result of a failed fusion between the septum primum (*arrows*) and the interatrial groove (IAG), which consists of a superior (S) and inferior (I) part. There virtually never is a volume overload in these patients because the transatrial flow is limited and oblique to the interatrial septum, due to the orientation of the septum primum flap. This feature distinguishes a PFO from other atrial septal defects where the flow direction is perpendicular to the axis of the interatrial septum. *LA* left atrium, *RA* right atrium

**Fig. 9:**
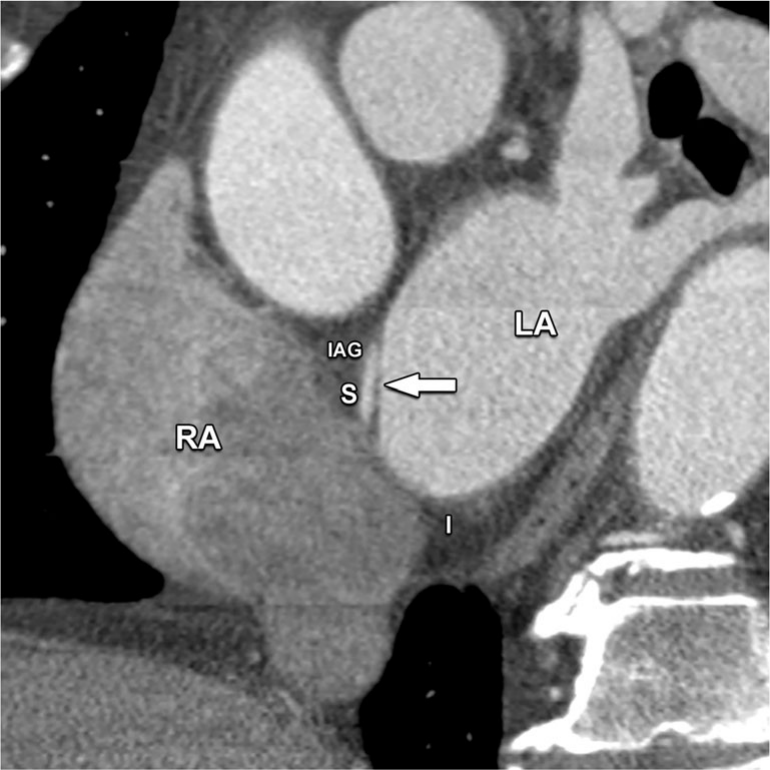
Oblique sagittal post-contrast CT image in a patient with an incidentally found probe PFO at the level of the superior part (S) of the interatrial groove (IAG). Similar to a PFO a septum primum flap (*arrow*) is present. However, in this entity functional closure of the foramen ovale has occurred and hence, no interatrial communication exists, in contrast to a real PFO. *I* inferior part of the interatrial groove, *LA* left atrium, *RA* right atrium

Reflecting the embryologic purpose of arteriovenous communication, a PFO has a vertical cranio-caudal orientation in the direction of the inferior vena cava, best seen on axial images or oblique images perpendicular to the atrial septum. Both the OP and secundum flap can then be identified, surrounded by dense intravenous contrast, as demonstrated in Fig. 8. A variable amount of dense contrast can often be seen flowing into the right atrium. Incomplete fusion does not necessary imply that an interatrial shunt exists, as the septum can be functionally closed with still identifiable septum flaps. This anatomic variation is called a probe PFO (Fig. 9).

PFO’s are mostly clinically silent, frequently seen during cardiac CT examination performed for unrelated reasons [8]. When large, they can also be detected on non-ECG gated CT examinations [13]. In asymptomatic patients with an incidental PFO, no medical or interventional therapy is advised. Furthermore, a potential benefit of having a PFO is facilitation of transseptal catheter passage in radiofrequency ablation procedures. The criteria for PFO closure are not standardized. Whether transcatheter PFO closure is considered superior to medical therapy in reducing the risk of recurrent stroke is currently the subject of intense debate and research [14, 15].

### Ostium primum defect

**Fig. 10:**
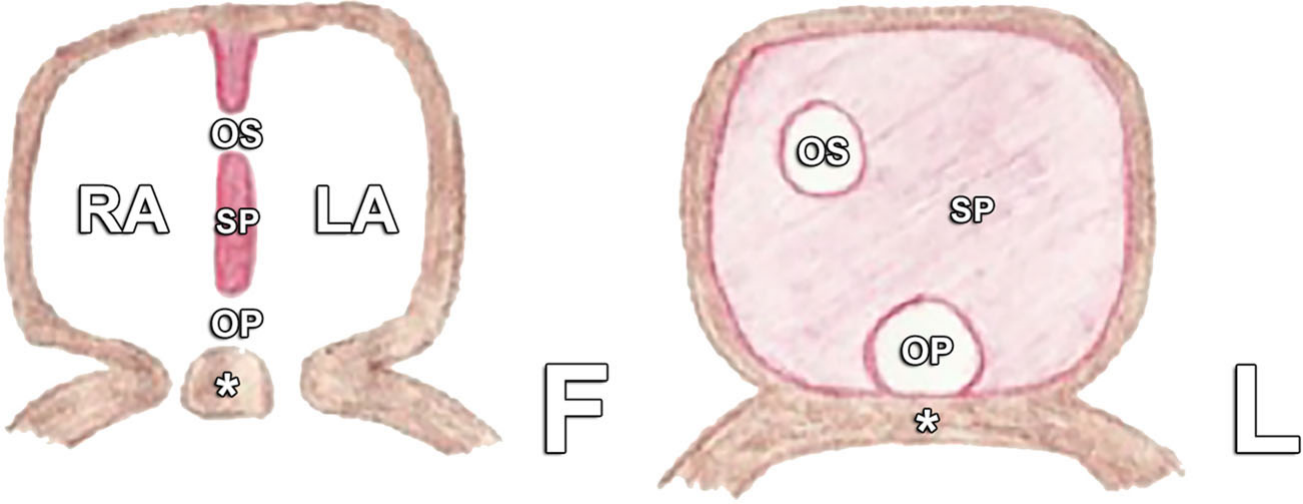
In normal embryological development, the OP closes and fuses with the endocardial cushions. The ostium secundum appears simultaneously, as illustrated in Fig. 2. An OP defect occurs when the OP fails to close and fuse with the endocardial cushions (*asterisk*), shown in frontal (F) and lateral (L) view. *LA* left atrium, *RA* right atrium, *SP* septum primum, *OS* ostium secundum

An OP defect is a less common type of ASD, accounting for 15 % of ASDs. They occur in the lower portion of the atrial septum near the AV valves. The embryological basis of this defect is shown in Fig. 10. In the presence of an OP defect, one should therefore always look for an associated valve defect, most frequently a cleft in the anterior leaflet of the mitral valve.

**Fig. 11:**
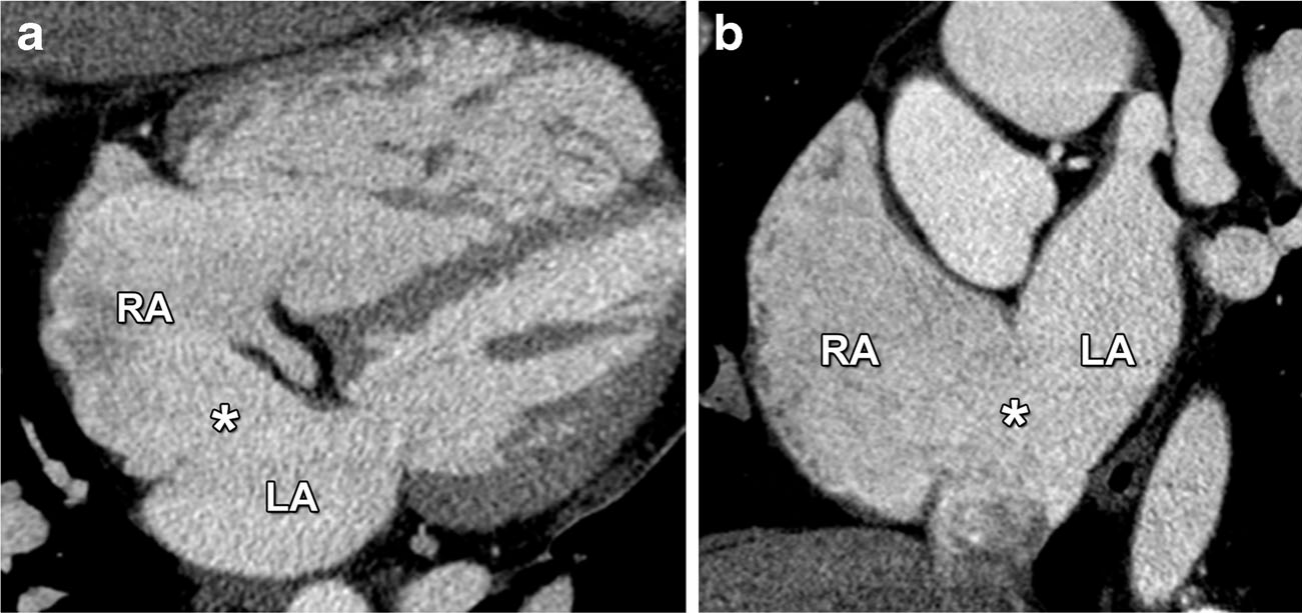
Four-chamber view (**a**) and short-axis (**b**) contrast-enhanced CT images showing a large OP defect (*asterisk*) allowing a communication between the left atrium (LA) and right atrium (RA). These defects are located in the part of the interatrial septum adjacent to the AV valves. Because OP defects are often large and lack a septal flap as in a patent foramen ovale, a shunt-induced volume overload with subsequent enlargement of the RA and ventricle can be seen

Since OP defects are often large they can easily be depicted on CT, revealing a large shunt flow, typically perpendicular to the interatrial septum. Oblique CT images allow detailed evaluation of the anatomy, such as the relationship with the AV valves and the absence of a flap, as illustrated in Fig. 11. As in all types of ASDs, surgical closure of the defect is indicated in those patients with a hemodynamically significant shunt that causes enlargement of the right heart structures. Suspicion of paradoxal embolism in the absence of other causes is also a valid indication for defect closure [16, 17].

### Ostium secundum defect

**Fig. 12:**
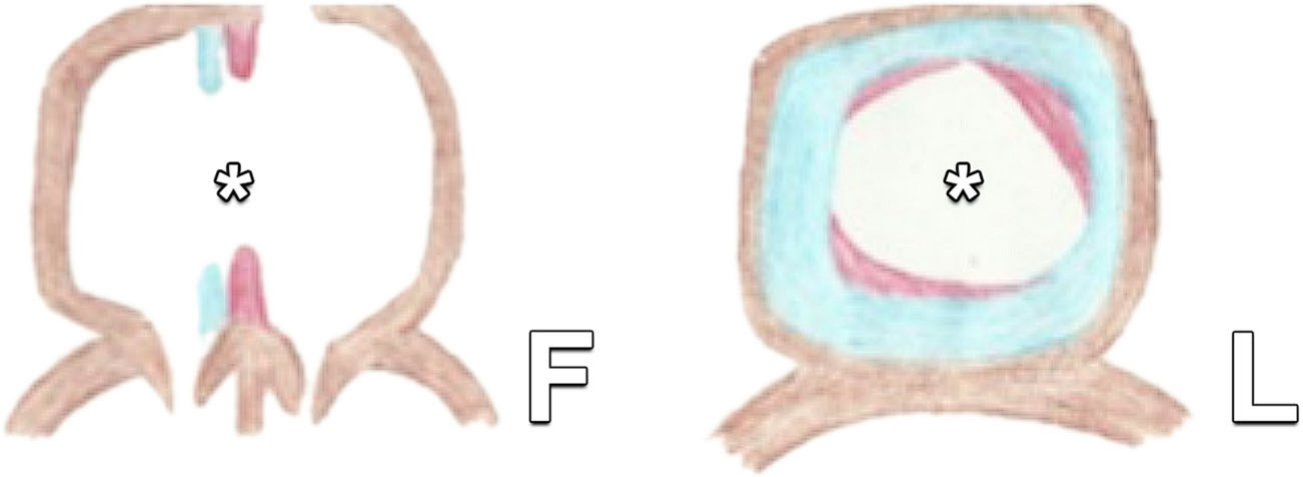
In normal embryological development, resorption of the septum primum at the ostium primum and formation of the foramen ovale in the septum secundum occur simultaneously, as illustrated in Fig. 2. In some cases, excessive resorption of the septum primum (drawn in *red*) or inadequate development of the septum secundum (drawn in *blue*) or a combination of both occurs. This leads to a larger than normal defect centrally in the interatrial septum at the future fossa ovalis, called an ostium secundum defect (*asterisk*). *F* frontal view, *L* lateral view

**Fig. 13:**
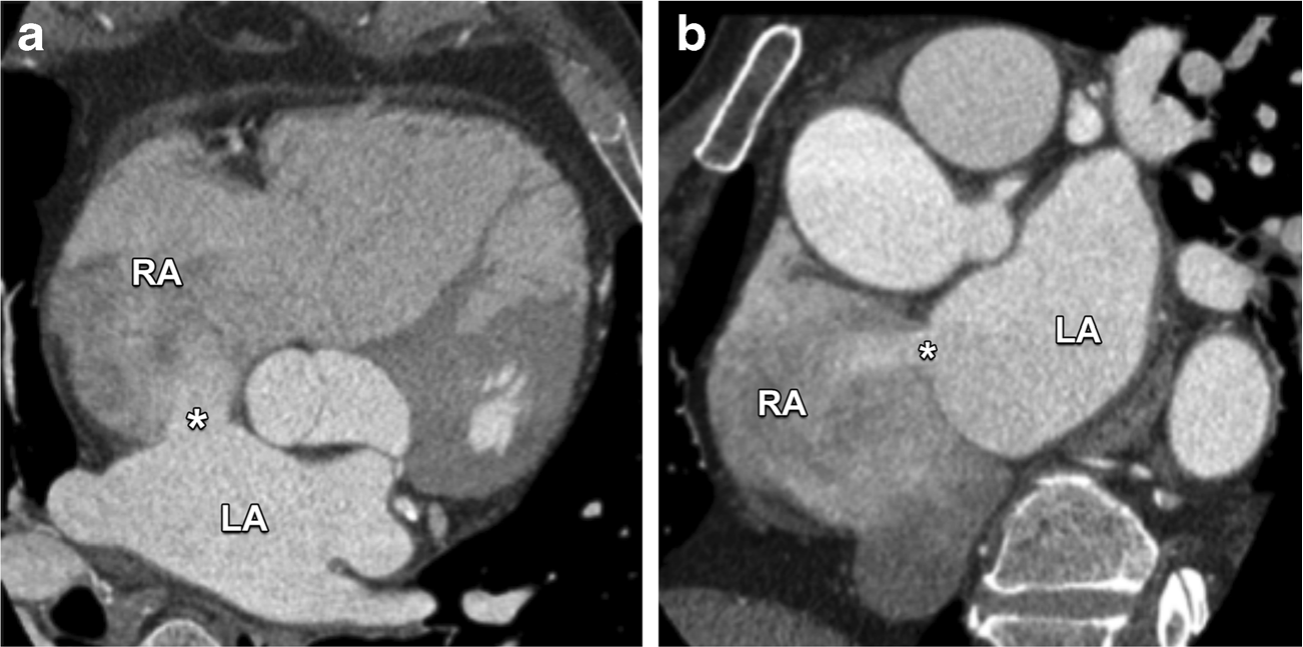
Axial (**a**) and coronal (**b**) contrast-enhanced CT images clearly depict an ostium secundum defect (*asterisk*) at the fossa ovalis with shunting of contrast-enhanced blood from the left atrium (LA) to the right atrium (RA). Contrary to a patent foramen ovale, the flow is perpendicular to the axis of the interatrial septum because of the absence of an overlapping septal flap. In large defects like in this case, this can lead to volume overload and dilatation of the right atrium and ventricle

Ostium secundum defects are the most frequent type of ASD, accounting for 75 % of all cases. They are found in the region of the fossa ovalis. The embryological basis of this ASD is illustrated in Fig. 12. Contrary to a PFO, an overlapping septal flap is lacking. As in OP defects, a shunt flow perpendicular to the axis of the interatrial septum is seen, in contrast to the more oblique cranio-caudal flow of a PFO, as demonstrated in Fig. 13.

Small defects are often asymptomatic and usually do not require treatment. Large defects, however, can initially cause volume overload of the right heart, eventually leading to pressure overload with dilatation of the right atrium and ventricle. As a consequence, right heart failure, pulmonary hypertension and right-to-left shunting may render this type of ASD to become symptomatic, usually in the fifth decade. This can be prevented by shunt closure, either surgically or endovasculary depending on the characteristics of the defect. Large defects and defects close to the edge of the interatrial wall are less suited for the endovascular approach since most closing devices cannot be anchored properly [16, 17].

### Sinus venosus defect

**Fig. 14:**
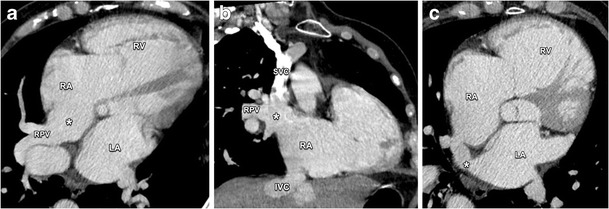
Axial (**a**) and coronal (**b**) contrast-enhanced CT images illustrate a sinus venosus defect (*asterisk*), located eccentrically in the interatrial septum near the entry of the super vena cava (SVC) into the right atrium (RA). Instead of draining into the left atrium (LA) the right pulmonary veins (RPVs) conjoin with the superior vena cava into the RA. There is also interatrial communication through a short vein segment (*asterisk* in **c**). The anomalous pulmonary venous return leads to a volume overload and secondary dilatation of the RA and ventricle. *IVC* inferior vena cava

**Fig. 15:**
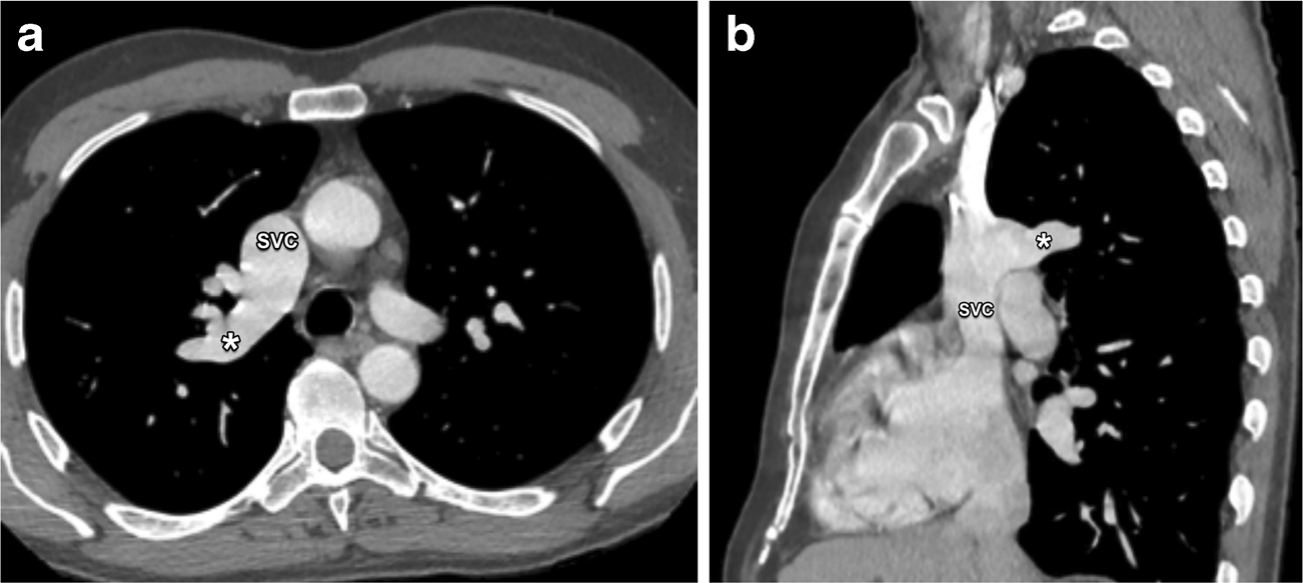
Axial (**a**) and oblique sagittal (**b**) post-contrast CT images of a patient with anomalous return of the right upper pulmonary vein (*asterisk*) in the superior vena cava (SVC)

Sinus venosus defects represent 4–11 % of atrial septum defects [18]. The name is derived from an abnormal absorption of the embryologic sinus venosus into the right atrium. In this defect, communication exists between one or more of the right pulmonary veins and the junction of the superior vena cava and the right atrium, called the superior vena cava type, as demonstrated in Fig. 14. Sometimes, only the superior vena cava is involved (Fig. 15). In fewer cases, this occurs just above the junction of the inferior vena cava and the right atrium (inferior sinus venosus defect) [19]. The presence of a sinus venosus defect may lead to volume and pressure overload of the right heart. Multiplanar CT reconstructions are very useful in the assessment of the complex anatomy of this type of ASD. Sinus venosus defects with significant hemodynamic impact need surgical correction to prevent further right heart failure and pulmonary hypertension [16, 17].

## Ventricular shunts

A ventricular septum defect (VSD) is an abnormal opening in the VS [5], allowing communication between the ventricular cavities. In children, VSDs are the second most common CHD (34 %) with a birth incidence of 2.62 per 1000 live births [1]. Most of these are small and spontaneously resolve in childhood, the majority within the 1 year after birth [20]. VSDs may also be acquired after acute myocardial infarction, chest trauma, cardiac interventions, or endocarditis.

In the adult population, the prevalence is estimated to be 0.3 per 1000 [21], making it the second most common form of CHD after the bicuspid aortic valve.

### Membranous VSD

**Fig. 16:**
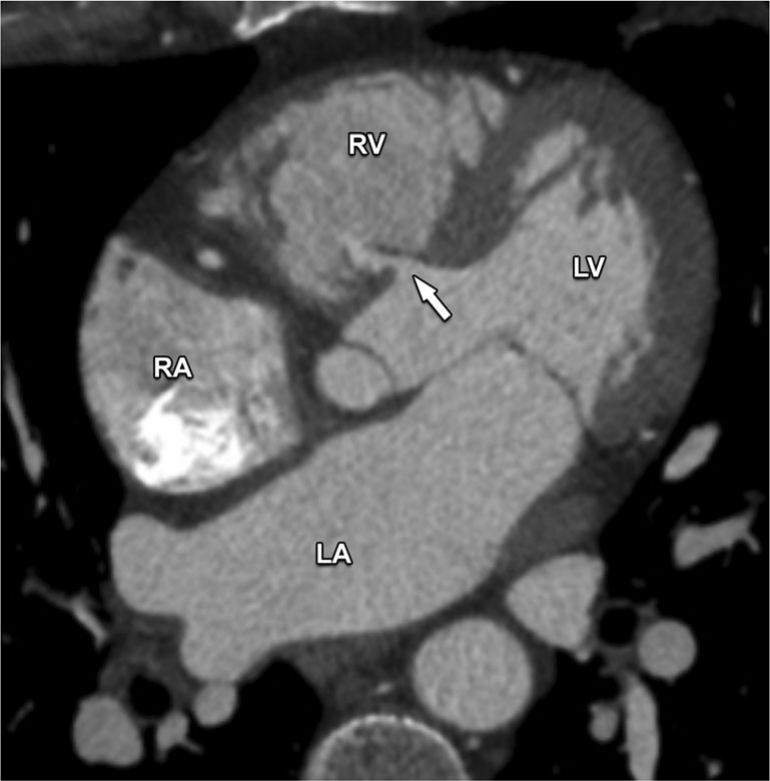
This axial contrast-enhanced CT nicely illustrates a defect in the membranous portion of the IVS (*arrow*). This defect permits a communication between the ventricular cavities, and causes blood shunting from the high pressure left systemic circulation to the low-pressure pulmonary circulation. The magnitude of the shunt is influenced by the size of the defect, the pressure in right ventricle (RV) and left ventricle (LV) and the pulmonary and systemic vascular resistance. *RA* right atrium, *LA* left atrium

Membranous VSDs are the most common type of VSD, accounting for 70–80 % of all cases. It is located in the upper part of the VS directly below the aortic valve and behind the septal leaflet of the tricuspid valve (Fig. 16). Because of their proximity to the defect regurgitation of these valves may occur.

There are two main subtypes, a true defect that is confined to the membranous septum alone and defects that extend into the muscular septum. Untreated VSDs in adults are almost always small defects with little hemodynamic load on the heart, and no risk of pulmonary vascular disease. Their only risk is infective endocarditis or arrhythmias. The proportion of VSDs that are large and hemodynamically significant is assumed to form 20 % of all VSDs [22]. In large defects, there is usually a volume overload of the right ventricle, the pulmonary circulation and eventually the left atrium and left ventricle. If untreated, this leads to a left ventricular dilatation, systolic dysfunction and heart failure. Eventually, even pulmonary vascular disease and right-to-left shunting (Eisenmenger syndrome) will develop [5]. Most patients with these large defects born before 1960 would not have had effective surgical treatment and very few of them would be alive today. Therefore, those adults seen today are virtually all postoperative [5, 23].

### Muscular VSD

**Fig. 17:**
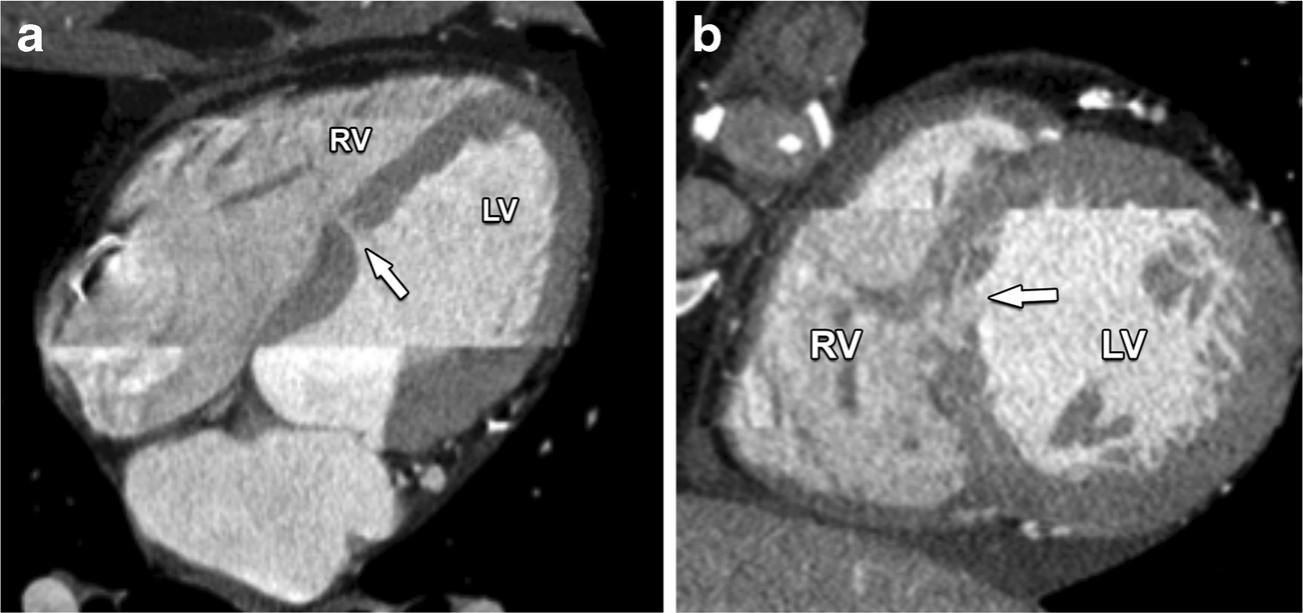
Four-chamber view (**a**) and two-chamber view (**b**) of contrast-enhanced CT in a patient with a muscular VSD. The defect (*arrow*) is confined to only the muscular part of the IVS. Like in membranous VSD, a large defect can lead to congestive heart failure or pulmonary vascular disease. *RV* right ventricle, *LV* left ventricle

These defects are confined to the muscular septum and account for 20 % of VSDs. The apical two thirds of the muscular septum is most frequently involved (Fig. 17). Multiple small defects (“Swiss cheese” septum) can occur and have the same hemodynamic effects as a single large defect [5]. Small and moderate VSDs in children frequently close entirely or sufficiently within the first 2 years making intervention unnecessary. They are often asymptomatic and have an excellent prognosis. When symptoms of heart failure or pulmonary vascular disease occur in neonates, often in case of large VSDs, surgical correction is recommended. Patients with an unclosed VSD are at risk for endocarditis and need a life-long prophylaxis. Adults with irreversible pulmonary damage and Eisenmenger syndrome are not eligible for closure of the defect, because right heart failure can be induced. Large defects with normal pulmonary vascular resistance, on the other hand, can still be treated with repair of the defect [24].

### Ventricle septum diverticulum

**Fig. 18:**
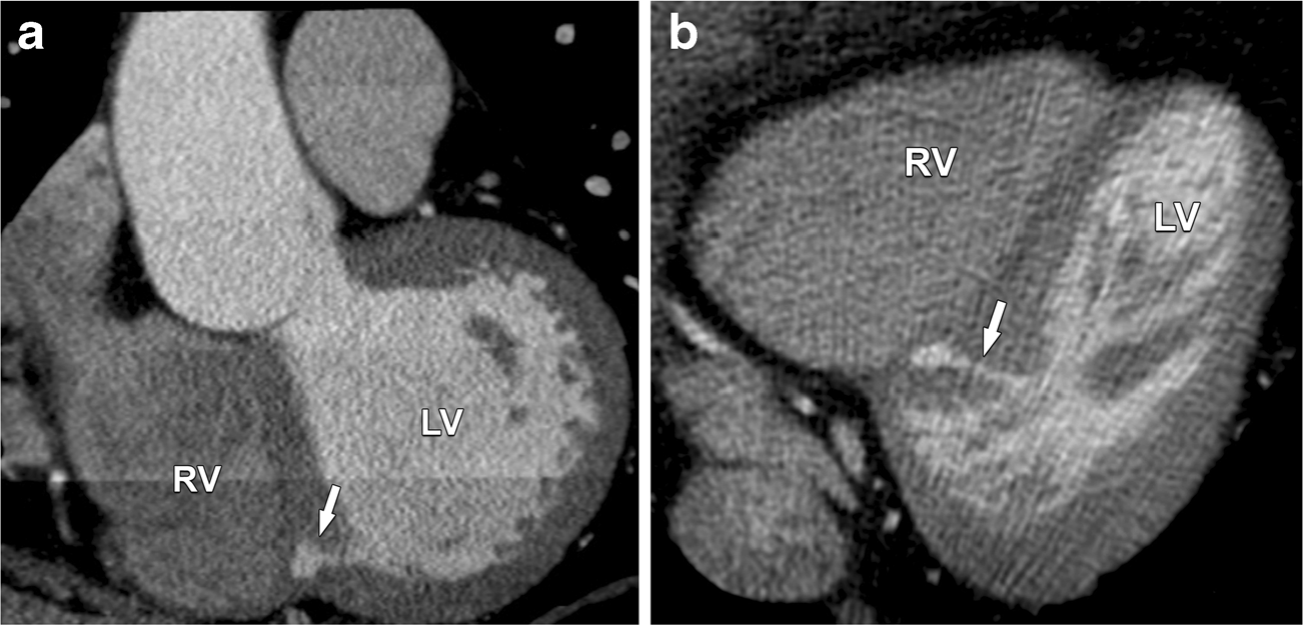
Coronal oblique (**a**) and axial (**b**) contrast-enhanced CT nicely show a ventricular septum diverticulum (*arrow*). There is a defect originating from the left ventricle (LV) extending in the muscular interventricular septum without reaching the right ventricle (RV). This entity has no hemodynamic importance but can increase the risk of endocarditis

Ventricle septum diverticulum is not a true VSD but it can be a mimicker, so it is worth mentioning. This is a rare congenital malformation that is defined as an out pouching of one of the ventricles into the interventricular septum, as shown in Fig. 18 [25].

### Rare VSDs

A supracristal VSD is a rare type (5–8 %) and is located beneath the aortic and pulmonic valves and connects the aorta with the right ventricular outflow tract. It is often associated with aortic valve prolapse. A second uncommon VSD (8–10 %) is situated posterior to the septal leaflet of the tricuspid valve. It is usually associated with an AV septal defect or anomalous insertion of the chordae tendinae of the AV valves.

## Imaging after intervention

**Fig. 19:**
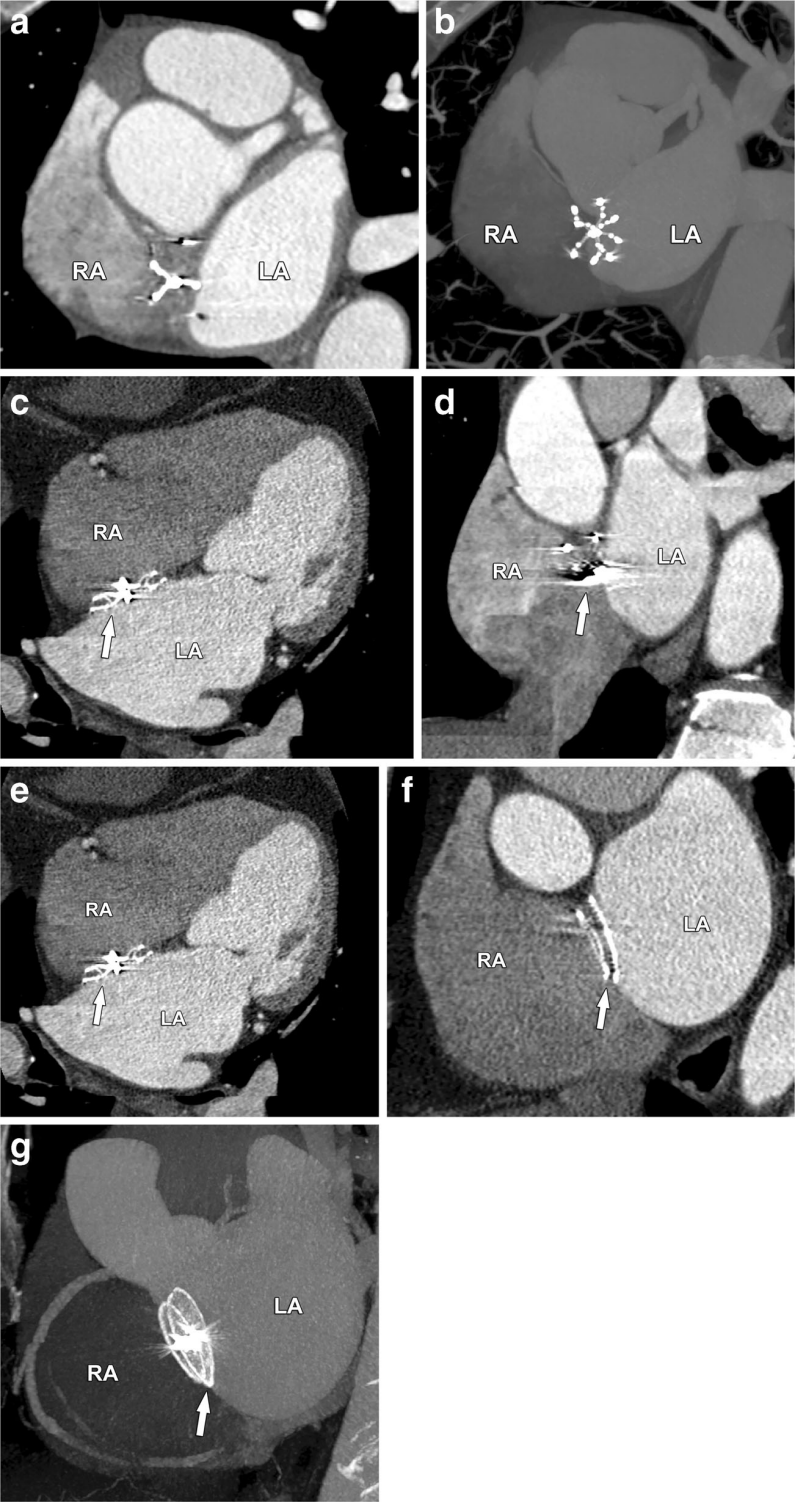
Different atrial septal occluder devices are shown: the Biostar© device (Organogenesis, Canton, MA, USA) in figures **a**–**b**, the Premere© device (St Jude Medical, St. Paul, MN, USA) indicated by *arrows* in Figures **c**–**d** and the Amplatzer© device (AGA Medical, Plymouth, MN, USA) indicated by *arrows* in Figures **e**–**f**. Difference in architecture between the devices can clearly be appreciated as well as a difference in artifact production, most pronounced in the Premere device. *RA* right atrium, *LA* left atrium

**Fig. 20:**
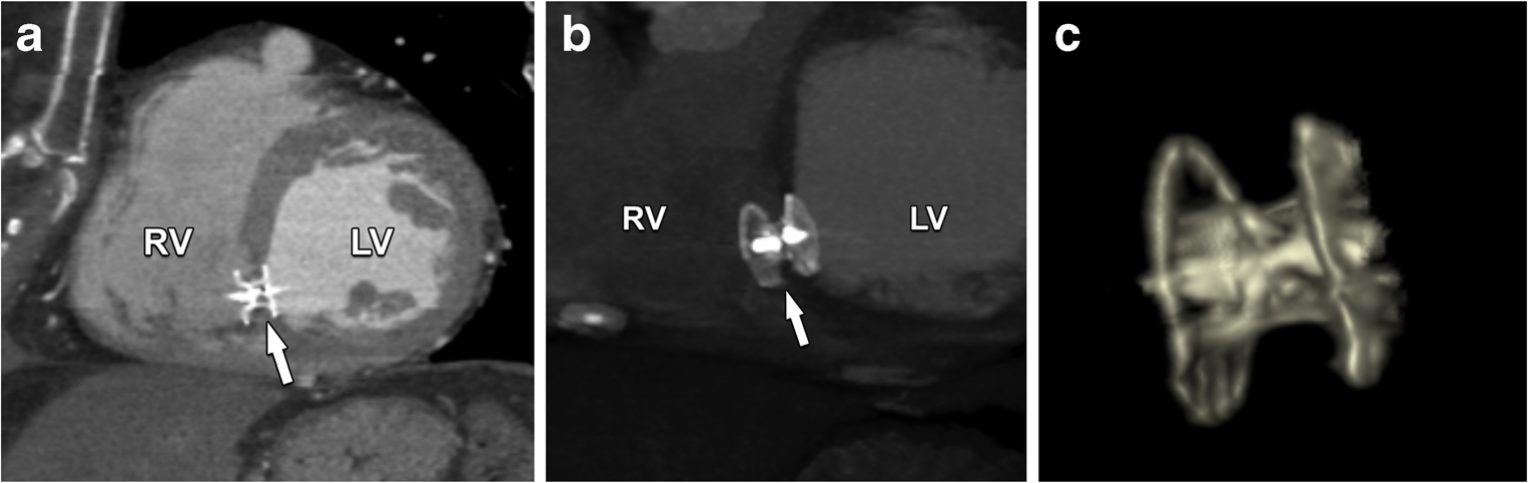
Two-chamber view of contrast-enhanced CT (**a**), maximum intensity projection (MIP) (**b**) and volume-rendered (**c**) images of a VSD closure device. *RV* right ventricle, *LV* left ventricle

Different devices are available today for closure of clinically significant intracardiac shunts. They not only differ in architecture but also in imaging compatibility, such as artefact production on CT. This is nicely illustrated in three different ASD closure devices in Fig. 19. Devices used for VSD closure are comparable with those used in ASD closure (Fig. 20). The choice of device and access route depends largely on the location and morphology of the shunt to be treated. Transoesophageal echocardiography remains the golden standard in this pre-procedural assessment, but CT is found to give equal information [8, 9]. Transoesophageal and transthoracic echocardiography, however, remain the reference standard for detection and follow-up of residual shunts [26, 27]. In post-procedural follow-up too, CT can still play an important role, i.e. in detecting complications at the site of intervention but also along the access route, e.g. pseudoaneurysm formation at the puncture site.

## Conclusion

Noninvasive cardiac CT imaging is becoming increasingly common in daily practice. But there is more to see than just the coronary arteries. Clinically important structural heart defects can be detected as well. Knowledge of this kind of pathology is the key to detection, which eventually increases the value of the radiological report. In this article, we give an overview of common types of congenital disease, the AV shunts. We try to emphasize which defects are clinically important and which are insignificant.
